# Psychological capital appreciation as a mediator between resilience and burnout among ICU nurses

**DOI:** 10.3389/fpubh.2025.1551725

**Published:** 2025-04-16

**Authors:** Rui Zhang, Mei Shan, Yanling Yin, Yanshuo Wu, Peng Gao, Yan Xin, Kangkang Shen

**Affiliations:** ^1^Department of Intensive Care Unit, The Fourth Hospital of Hebei Medical University, Shijiazhuang, China; ^2^Department of Intensive Care Unit, The Third Hospital of Shijiazhuang, Shijiazhuang, China

**Keywords:** burnout, intensive care unit, nurse, psychological capital appreciation, psychological resilience

## Abstract

**Objective:**

This study investigated the mediating effects of psychological capital appreciation in the relationship between psychological resilience and burnout among ICU nurses. The findings aim to provide an objective reference for hospitals to enhance the occupational health of ICU nurses.

**Methods:**

A cross-sectional questionnaire survey was administered to 150 ICU nurses employed at 20 tertiary hospitals across Hebei Province. Stratified random sampling was employed in the sampling methodology, with strata defined by hospital size and ranking. Subsequently, ICU nurses were randomly selected within each stratum to enhance the representativeness of the sample.

**Results:**

(i) Among the 150 nurses, the psychological resilience score was (68.701 ± 14.549), resilience was (63.547 ± 14.020), and burnout score was (65.095 ± 18.461). (ii) The analysis revealed that psychological capital appreciation mediated the relationship between psychological resilience and burnout, with a mediation effect size of 0.597, accounting for 79% of the total effect. (iii) Psychological resilience did not directly affect job burnout (direct effect = 0.148, 95% CI includes 0, *t* = 0.864, *p* = 0.389), but indirectly reduced burnout by enhancing PCA. The indirect effect was significant (indirect effect = 0.601, 95% CI excludes 0, *z* = 6.073, *p* = 0.000), with a total effect size of 0.748 (95% CI excludes 0, *z* = 8.486, *p* = 0.000).

**Conclusion:**

(i) Psychological capital appreciation (PCA) plays a complete mediating role between resilience and job burnout of ICU nurses. (ii) The overall incidence of job burnout among nurses is high, while the levels of resilience and psychological capital appreciation are at a low to medium level, which need to be improved urgently.

## 1 Introduction

Psychological capital appreciation (PCA) refers to a positive psychological state exhibited by individuals during their growth and development. It serves as a fundamental psychological resource distinct from human capital and social capital, acting as a catalyst for work motivation and an important emotional asset (1). PCA is systematically evaluated through the assessment of self-efficacy, hope, resilience, and optimism (2, 3).

Psychological resilience is the ability to respond and adapt to changes in the external environment. It reflects the capacity of an individual to recover from negative states and is an index of their flexibility in adapting to environmental changes (4, 5). In essence, psychological resilience manifests as a mental toughness that enables individuals to remain positive in the face of setbacks and difficulties (6). This ability is not entirely innate but can be developed and strengthened through challenging experiences (7).

Burnout is primarily defined as the physical and psychological harm caused when the workload of an individual exceeds a certain threshold. Originally proposed by Freudenberger in 1974, burnout is characterized by emotional exhaustion and is most common in the helping profession. It represents a suboptimal mental health state (8). Notably, medical industry research indicates that burnout varies across departments, with certain specialties exhibiting distinct patterns. For instance, medical staff in emergency departments and intensive care units (ICU) experience significantly higher levels of burnout compared to those in general internal medicine or surgical wards (9).

ICU nurses have high work intensity and pressure (10), and their mental health is closely related to nursing quality and patient safety, which has attracted much attention. Psychological resilience can help them cope with negative emotions, and psychological capital depletion reflects the depletion of psychological resources, and the two are closely related. In the existing literature, the research on psychological resilience mainly focused on the concept and general measurement (11), and the special influencing factors and mechanisms of ICU nurses were insufficient. Although the study of psychological capital consumption mentioned the influence of work stress, there was a lack of in-depth analysis of ICU nurses. Moreover, few studies have analyzed the relationship between them from the perspective of psychological capital. In the ICU environment, how psychological capital plays a mediating role between psychological resilience and psychological capital consumption is still blank.

In the past, some scholars have conducted in-depth analyses on the relationship between individual psychological traits and occupation-related states. As the key psychological adjustment ability of individuals in the face of adversity, psychological resilience has a significant impact on psychological capital. Shen et al. (12) believe that strong psychological resilience can help individuals accumulate and improve psychological capital in stressful situations, and lay a solid foundation for them to cope with work and life challenges. The relationship between psychological capital and job burnout has also attracted academic attention. Sun et al. (13) believe that individuals with a higher level of psychological capital are more proactive in work and can effectively resist the invasion of job burnout. High psychological capital can promote individuals to look at work pressure with a more optimistic attitude, enhance their ability to cope with career difficulties, and thus reduce the possibility of job burnout.

The high-stress nature of ICU nursing can have severe consequences not only for the nurses’ personal wellbeing but also for the quality of patient care. If nurses experience burnout, it may lead to decreased job satisfaction, increased turnover rates, and ultimately, compromised healthcare delivery. By conducting in-depth research on this specific group, we can better understand how psychological resilience and positive coping strategies interact to mitigate burnout. This knowledge can then be translated into targeted interventions and support systems tailored to the needs of ICU nurses.

This study used a structural equation model to explore this mediating effect for the first time, aiming to fill the literature gap, enrich the theory of nursing psychology, provide a basis for medical institutions to formulate mental health intervention strategies for ICU nurses, and improve the mental health and occupational wellbeing of nurses.

## 2 Participants and methods

### 2.1 Research participants

An online questionnaire survey was administered to 150 ICU nurses from 20 tertiary hospitals, including the Fourth Hospital of Hebei Medical University and the Third Hospital of Hebei Medical University. The use of stratified random sampling based on hospital size and rank was based on the following considerations. Hospital scale is closely related to medical resources, the number of patients and the complexity of nursing. ICU resources in large hospitals are rich, and patients’ conditions are complex, so nurses need to master more complex skills and face more challenges. Through this stratification, we can comprehensively cover different levels of ICU nursing environment, make the sample more representative, and then accurately reflect the real situation of ICU nurses, and enhance the universality and reliability of the study. The whole process of answering questions took the form of an anonymous answer sheet, and a strict privacy protection mechanism was constructed. A total of 136 valid responses were obtained, yielding an effective response rate of 91.33%. In this study, the questionnaire data were initially screened by statistical software, and the questionnaires that were not logical (such as all option A, the whole questionnaire answer less than 1 min, etc.) were marked. Then researchers manually reviewed these marked questionnaires, finally determined the list of invalid questionnaires, and deleted the invalid questionnaires. The inclusion criteria were registered nurses with at least 1 year of clinical experience in an ICU setting, who provided informed consent and voluntarily participated in the study. Exclusion criteria encompassed nurses who had resigned or transferred out of the ICU, those unable to complete the survey due to studies or further education, and nurses with a past or present history of severe physical or mental illness.

Elimination criteria involved responses with excessively long or short completion times and those with inconsistent or patterned answers showing evident logical contradictions.

### 2.2 Research tools

Basic demographic information, including sex, age, professional title, job title, and other related data were collected.

After data collection, a comprehensive reliability and validity test was conducted based on the data obtained. At the same time, at the end of the paragraph introducing each scale, the test results and related conditions were explained in detail to ensure the transparency and traceability of the research process and data quality.

Burnout scale: This study employed the Chinese version of the burnout scale revised by Chaoping et al. (8). The scale consists of 15 items scored on a seven-point Likert scale. The total score is calculated by summing the scores for all items with the final evaluation derived by multiplying the average score per item by 20, with 0–50 points indicating good condition; 50–75 points indicating mild burnout state; 75–100 points indicating severe burnout state; and more than 100 points indicating extremely severe burnout state. The scale demonstrated high reliability with an *α* coefficient of 0.949 and excellent validity with a KMO value of 0.908 (8).

Nurse psychological capital appreciation scale: This scale was originally developed by Luthans et al. (14). The simplified Chinese version, developed by Luo and He, was employed in this study to measure the PCA level of ICU nurses (15). The scale consists of four dimensions and 12 items scored on a 7-point Likert scale. The total score is obtained by summing the factor scores, with higher total scores indicating higher PCA. In this study, the reliability and validity of the collected and collated data were tested, and the results were consistent with those tested by scholars such as Luo (15), and this scale demonstrated excellent reliability with an *α* coefficient of 0.976 and high validity with a KMO value of 0.946.

Connor-Davidson resilience scale (CD-RISC): The Chinese version of the scale revised by Jiao Chunhui and Qu Qingrong was employed for this investigation (5). The scale consists of 10 items and is scored on a 7-point scoring scale. The total score is calculated by summing the factor scores, with higher scores indicating higher levels of psychological resilience. The Chinese version of the CD-RISC (10 items) is reliable, valid, easy to use, and is suitable for clinical settings (16).

### 2.3 Statistical analysis

Data collected from the survey were statistically analyzed using SPSS 26.0.

#### 2.3.1 Correlation analysis of resilience, PCA and job burnout

In this study, the Pearson method was used to conduct a Bayesian correlation analysis of resilience, PCA, and job burnout (see Table 1). Before analysis, normality and homogeneity of variance tests were performed. The Jarkue-Bera test was used to determine whether job burnout, psychological capital, and psychological resilience obey normal distribution. The results showed that the *p* values of each variable were greater than 0.05, and they obeyed normal distribution at the 0.05 significance level. At the same time, Levin’s homogeneity of variance test was performed. Taking job burnout as the benchmark, the *p* values of psychological capital and psychological resilience were greater than 0.05, which was consistent with the hypothesis of homogeneity of variance.

**Table 1:** Results of the mediating effect test.

Item	Symbol	Significance	Effect	95% CI		SE value	*z* value /*t* value	*p* value	Conclusion
				Lower limit	Upper limit				
Psychological resilience = > Psychological capital appreciation = > Burnout total score	a*b	Indirect effects	0.601	0.276	0.662	0.099	6.073	0.000	Full mediator
Psychological resilience = > Psychological capital appreciation	a	X= > M	0.842	0.762	0.922	0.041	20.676	0.000	
Psychological capital appreciation = > Burnout total score	b	M= > Y	0.713	0.366	1.060	0.177	4.024	0.000	
Psychological resilience = > Burnout total score	c’	Direct effects	0.148	−0.188	0.483	0.171	0.864	0.389	
Psychological resilience = > Burnout total score	c	Total effects	0.748	0.575	0.921	0.088	8.486	0.000	

It presents the various effect values, confidence intervals, statistics, and *p*-values of the mediating effect in a horizontal format to determine the situation of the mediating effect.

To enhance the robustness of the study, a non-parametric test (Kruskal Wallis test) was performed, and the grouping variable was burnout. The results showed that the asymptotic significance of both resilience and psychological capital was less than 0.05, indicating that there were significant differences in resilience and psychological capital among the different burnout groups.

Pearson correlation analysis showed that the confidence intervals and effect sizes of ICU nurses’ burnout, resilience, and psychological capital appreciation studies were statistically significant. The 95% confidence interval of the correlation between job burnout and psychological capital appreciation was [0.535, 0.733], and the mean value was 0.645; the confidence interval of psychological resilience was [0.466, 0.687], and the mean value was 0.580; the confidence interval of psychological capital appreciation and psychological resilience was [0.823, 0.905], and the mean value was 0.867. This indicates that at the 95% confidence level, burnout is significantly positively correlated with psychological capital appreciation and resilience, and the correlation is moderate or above, and psychological capital appreciation is strongly positively correlated with resilience.

#### 2.3.2 Analysis of mediating effect

**Figure 1:**
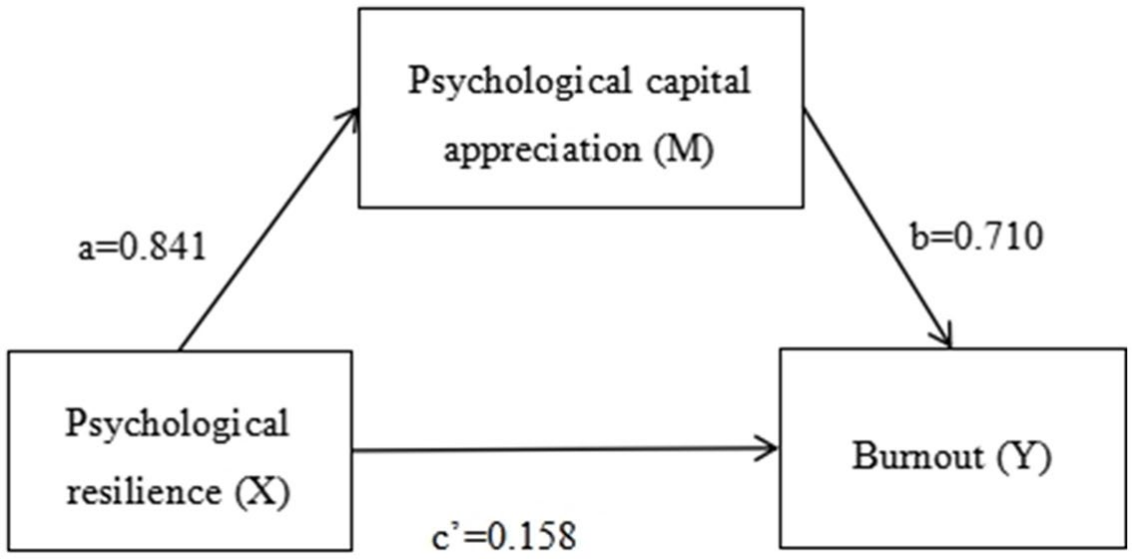
Diagram of the mediating effect model. Psychological resilience has a direct positive effect on burnout (with a coefficient of 0.158) and also has an indirect positive effect on burnout through psychological-capital appreciation (with coefficients *a* = 0.841 and *b* = 0.710).

According to Tables 1, 2, and Figure 1, resilience was positively correlated with psychological capital appreciation, the effect value was 0.842, the 95% confidence interval did not include 0, *z* value was 20.676, *p* value was 0.000; Psychological capital appreciation had a significant negative relationship with the total score of job burnout (effect value 0.713), 95% confidence interval did not include 0, *z* value 4.024, *p* value 0.000; Psychological capital appreciation played a complete mediating role between psychological resilience and job burnout score, the indirect effect value was 0.601, the 95% confidence interval did not include 0, *z* value was 6.073, *p* value was 0.000; The direct effect value of resilience on the total score of job burnout was 0.148, the 95% confidence interval included 0, *t* value 0.864, *p* value 0.389, and the total effect value was 0.748, the 95% confidence interval did not include 0, *z* value 8.486, *p* value 0.000, and was significant. The overall effect is mainly contributed by the indirect effect through psychological capital appreciation.

**Table 2:** Test of the mediating effect model.

Variables	Burnout	Psychological capital appreciation	Burnout
Constant	13.593	5.697*	9.531
	(2.199)	(1.995)	(1.604)
Psychological resilience	0.748**	0.842**	0.148
	(8.486)	(20.676)	(0.864)
Psychological capital appreciation	No	No	0.713**
	No	No	(4.024)
Sample size	136	136	136
*R* ^2^	0.350	0.761	0.420
Adjusted *R*^2^	0.345	0.760	0.411
*F* value	*F*(1.134) = 72.017, *p* = 0.000	*F*(1.134) = 427.492, *p* = 0.000	*F*(2.133) = 48.185, *p* = 0.000

This table provides the constant terms, regression coefficients, sample size, determination coefficients, etc. of the mediating effect model, evaluating the goodness of fit of the model and the significance of variables.

*p < 0.05, **p < 0.001, *t*-value in parentheses.

## 3 Results

### 3.1 Demographic data analysis

According to Table 3, the demographic data of 136 subjects were analyzed. There were 32 males (23.5%) and 104 females (76.5%), with the majority of females. In terms of marital status, 107 were married, accounting for 78.7%, 28 were unmarried, accounting for 20.6%, and 1 was divorced, accounting for 0.7%. In terms of educational background, 127 (93.4%) had a bachelor’s degree, 1 (0.7%) had a master’s degree or above, and 8 (5.9%) had junior college education. In terms of ICU working years, 1–15 years were more concentrated, with 37 workers (27.2%) for 1–5 years and 6–10 years, 41 workers (30.1%) for 11–15 years, 10 workers (7.4%) for less than 1 year, and 11 workers (8.1%) for more than 15 years.

**Table 3:** Results of demographic statistics.

Variables	Categories	Frequency	Percentage	Valid Percentage	Cumulative Percentage
Gender	Male	32	23.50%	23.50%	23.50%
Gender	Female	104	76.50%	76.50%	100.00%
Marital status	Married	107	78.70%	78.70%	78.70%
Marital status	Unmarried	28	20.60%	20.60%	99.30%
Marital status	Divorced	1	0.70%	0.70%	100.00%
Educational attainment	Junior College	8	5.90%	5.90%	5.90%
Educational attainment	Undergraduate	127	93.40%	93.40%	99.30%
Educational attainment	Master’s Degree and Above	1	0.70%	0.70%	100.00%
Working years in ICU	Less than 1 year	10	7.40%	7.40%	7.40%
Working years in ICU	1–5 years	37	27.20%	27.20%	34.60%
Working years in ICU	6–10 years	37	27.20%	27.20%	61.80%
Working years in ICU	11–15 years	41	30.10%	30.10%	91.90%
Working years in ICU	More than 15 years	11	8.10%	8.10%	100.00%

### 3.2 Resilience, PCA, and burnout scale scores

According to Table 4, in the study of 136 ICU nurses, three scales were used for evaluation. The total score of the Conner-Davidson Resilience Scale was obtained by adding the scores of the factors, and the higher the score, the higher the psychological resilience. The average score of this study was 68.58 ± 14.54, which was in the middle level. The nurses’ psychological capital appreciation Scale obtained the total score by adding the scores of the factors, and the higher the score, the higher the PCA value. The average PCA score was 63.46 ± 14.03, which was a moderate level. Self-efficacy (12.08 ± 3.59), hope (17.00 ± 3.92), resilience (17.19 ± 3.72), and optimism (17.19 ± 3.71); The average score of each item was multiplied by 20 to obtain the total score. The grading standard was 0–50 points for good condition, 50–75 points for mild burnout, 75–100 points for serious burnout, and more than 100 points for extremely serious burnout. The reference grading standard was between 50 and 75, indicating a high level of job burnout, and the specific dimension scores were emotional exhaustion 19.47 ± 6.98, depersonalization 21.82 ± 6.92, and invalidity 23.62 ± 7.14, respectively. In general, the resilience and PCA of ICU nurses are at a medium level, but the level of job burnout is high. According to the corresponding relationship between the scores of the Maslach Burnout Scale and the grading standard, the data directly reflected that the subjects had experienced more significant job burnout, which may suggest that higher job burnout has an impact on the appreciation of psychological capital and then affects the level of psychological resilience. The specific relationship between the three is analyzed in the mediating effect.

**Table 4:** Scores of burnout, psychological resilience, and psychological capital appreciation (PCA) scores among ICU nurses.

Variables	*N*	Mean value	Standard deviation	Variance
Psychological resilience	136	68.59	14.54	211.49
PCA scores	136	63.46	14.04	197.03
Optimism	136	17.19	3.71	13.76
Resilience	136	17.19	3.73	13.904
Hope	136	17.00	3.93	15.48
Self-efficacy	136	12.08	3.59	12.89
Total burnout score	136	64.91	18.40	338.69
Emotional exhaustion	136	19.47	6.98	48.77
Depersonalization	136	21.82	6.92	47.93
Sense of personal fulfillment	136	23.63	7.14	51.02
Number of valid cases	136	None	None	None

This table lists in detail the statistical data such as the mean values, standard deviations, and variances of 136 ICU nurses on relevant psychological indicators and burnout dimensions, reflecting the central tendency and dispersion of the data.

### 3.3 Correlation analysis between psychological resilience, PCA, and burnout

Bayesian correlation analysis was performed using Pearson’s method (Table 5). In this study of ICU nurses’ job burnout, resilience, and psychological capital appreciation, Pearson method was used to carry out Bayesian correlation analysis.

**Table 5:** Correlation analysis between psychological resilience, psychological capital appreciation, and burnout in ICU nurses.

Posterior distribution characteristics of pairwise correlations^a^					
Variables 1	Variables 2	Data types	Burnout	Psychological capital appreciation	Psychological resilience
Burnout	Posterior distribution	Mode	No data	0.644	0.590
		Mean	No data	0.645	0.580
		Variance	No data	0.003	0.003
	95% confidence interval	Lower limit	No data	0.535	0.466
		Upper limit	No data	0.733	0.687
	*N*		136	136	136
Psychological capital appreciation	Posterior distribution	Mode	0.644	No data	0.872
		Mean	0.635	No data	0.867
		Variance	0.003	No data	0.000
	95% confidence interval	Lower limit	535	No data	0.823
		Upper limit	0.733	No data	0.905
	*N*		136	136	136
Psychological resilience	Posterior distribution	Mode	0.590	0.872	No data
		Mean	0.580	0.867	No data
		Variance	0.003	0.000	No data
	95% confidence interval	Lower limit	0.466	0.823	No data
		Upper limit	0.687	0.905	No data
	*N*		136	136	136

It presents the posterior distribution characteristics and confidence intervals of the pairwise correlations among the three, showing the degree of association between variables.

a Analytical hypothesis referring to prior research (*c* = 0).

We chose Bayesian correlation analysis for three main reasons. Bayesian analysis allows using prior knowledge from existing literature, which is beneficial, especially with small sample sizes, unlike traditional frequentist methods relying solely on current data. Also, it provides the posterior probability distribution of correlation coefficients, giving not only a point estimate but also a range of values and their probabilities, thus better understanding complex relationships like PCA’s mediating role, while traditional methods with just a single-value estimate and *p*-value are less informative. Moreover, as our data had non-normality challenging for traditional parametric methods, the flexibility of Bayesian methods, not strictly requiring normality assumptions, enables accurate data analysis without complex transformations that could distort relationships, leading to more reliable results.

In-depth analysis of the logical relationship between the three shows that ICU nurses with higher psychological resilience can better cope with work pressure and challenges, reduce the degree of job burnout, and are more inclined to actively seek solutions in the face of difficulties and promote the appreciation of psychological capital. Nurses with increased psychological capital have strong internal psychological strength, which can cope with high-intensity work with a more positive attitude and reduce job burnout.

In summary, job burnout was significantly positively correlated with psychological capital appreciation and psychological resilience, respectively, and psychological capital appreciation was strongly positively correlated with psychological resilience. This result provides key data support for understanding the occupational status and psychological characteristics of ICU nurses and provides key data support for formulating targeted intervention measures such as improving nurses’ psychological resilience training and enhancing psychological capital construction. It has important guiding significance to alleviate job burnout and improve job satisfaction and efficiency.

### 3.4 Analysis of mediating effects

According to Tables 1, 2, analysis shows PCA fully mediates the link between psychological resilience and burnout in ICU nurses. The mediating effect size of PCA is 0.597, making up 79% of the total effect. Further, as a mediator, PCA has a 100% effect size in this relationship. This indicates psychological resilience has no direct impact on burnout.

Psychological resilience helps ICU nurses recognize work stressors such as long hours and emotional strain. Instead of directly influencing burnout, it activates coping mechanisms, with PCA being key. Nurses use PCA’s positive problem-solving to manage workloads and balance work-life, like making efficient routines, thus reducing burnout.

The ICU’s complex, high-pressure environment means psychological resilience alone cannot fight burnout. PCA steps in as an intermediate. Through PCA’s positive reappraisal, nurses see patient-care challenges as growth opportunities, lowering burnout risks.

Psychologically, psychological resilience promotes the use of PCA. When faced with potential burnout, resilient nurses are more likely to use PCA, blocking the direct path from resilience to burnout. Resilience’s influence goes through PCA to determine burnout levels. In summary, PCA fully mediates this relationship, with psychological resilience having no direct effect on burnout.

## 4 Discussion

### 4.1 PCA and psychological resilience of ICU nurses

The PCA and psychological resilience levels among the surveyed ICU nurses were found to be moderate. The positive psychological state and inner psychological resilience of the ICU nurses surveyed were somewhat inhibited, which is closely related to the extremely heavy daily workload and day-and-night shifts that ICU nurses face. Currently, nurses in general ICUs deal with an increasing number of patients and diversified diseases, which places greater demands on their professional abilities and psychological resilience (17).

Previous studies have highlighted the critical role of PCA in fostering teamwork, enhancing team efficiency, and promoting effective collaboration among nurses (18). It is also correlated with the attitude of the nurses toward patients with improvements in PCA linked to higher care quality (19).

In terms of psychological resilience, studies have suggested that low resilience is a key factor leading to higher turnover rates among nurses (20). The turnover rate of ICU nurses is higher than that of other departments^.^ A higher turnover rate is detrimental to the overall quality of care in nursing teams, which can negatively affect the care and treatment of critically ill patients (21). It is worth noting that PCA is a modifiable key factor. Compared with some personal traits that are difficult to change in a short period of time, improving PCA through targeted interventions provides a practical way to improve the occupational health status of ICU nurses.

### 4.2 High levels of burnout among ICU nurses

Burnout levels observed among ICU nurses were notably high, indicating a critical situation that requires urgent attention. Burnout was characterized by three key dimensions: emotional exhaustion, depersonalization, and ineffectiveness. Emotional exhaustion refers to the depletion of emotional resources, resulting in physical fatigue, lack of enthusiasm, and motivation decline, often leading to frustration and emotional fatigue. Depersonalization manifests as disengagement, where nurses complete tasks passively, maintain emotional distance from colleagues, and avoid engagement with nursing managers. A sense of ineffectiveness indicates a low sense of self-worth among nurses.

The psychological stress associated with burnout poses significant risks to physical and mental health. For instance, Sullivan V and Hughes V et al. proposed that chronic burnout stress impairs multiple systems, including immune, circulatory, neuroendocrine, and central nervous systems (22). Prolonged exposure to burnout stress exacerbates functional decline, particularly in the immune system. This can eventually lead to cellular immunity depletion and trigger inflammation in the body through cytokine release (23). A number of studies have shown that job burnout is generally associated with decreased personal wellbeing and increased turnover intention in both Chinese and international nurses (20, 24). Cohen C, Pignata et al. proposed solutions from two perspectives: (1) establishing peer support systems and integrating mental health professionals within the nursing team to provide supportive care treatment; (2) encouraging relaxation techniques such as massage, acupuncture, and yoga to help reduce stress and promote wellbeing (25).

Compared with previous studies, there are discrepancies as well as consistencies. Harwood (4) have found that there is a correlation between psychological resilience and job burnout, and psychological capital plays a certain role in it, which is consistent with the results of this study, indicating that the relationship between psychological resilience, psychological capital and job burnout has certain common characteristics in nursing groups in different regions. Wang (9) pointed out that positive psychological capital played a mediating role in the relationship between nurses’ perceived organizational climate and job burnout, which was similar to the mediating role of psychological capital appreciation (PCA) between psychological resilience and job burnout in this study, indicating that psychological capital plays an important role in influencing nurses’ job burnout.

### 4.3 PCA as a full mediator between psychological resilience and burnout

PCA was found to fully mediate the relationship between psychological resilience and burnout. The psychological resilience score of ICU nurses not only predicts their burnout status but also has a profound impact on burnout through PCA, acting as a bridge between the two (26).

Psychological resilience (27), defined as the ability of an individual to resist stress and adapt to change, directly predicts burnout status. It provides predictive insight into future burnout risk, even before employment. This influence operates through PCA, which includes the optimism of an individual, positive outlook, and level of self-efficacy. Additionally, studies have suggested that a positive working environment can improve the psychological wellbeing of nurses, enhancing psychological resilience and reducing burnout (25). This in turn helps decrease the likelihood of depression and anxiety caused by burnout, allowing nurses to focus more on patient care (28, 29). By improving psychological resilience both PCA and burnout can be improved, leading to higher team efficiency and better occupational protection for nurses (30). This reduces the psychological damage from occupational exposure and minimizes somatic dysfunction caused by stress, ultimately improving the overall wellbeing of the nursing community (31).

Therefore, based on the results of this study, interventions in three key areas are recommended to address burnout effectively. First, enhance psychological resilience and stress resistance by cultivating higher levels of psychological resilience in ICU nurses (32). Second, incorporate psychological resilience evaluations during the recruitment process for new nurses. By selecting nurses with high levels of psychological resilience, the overall resilience and burnout levels of the ICU team can be improved. Third, create a positive and supportive atmosphere within the ICU department to improve its work environment (33). Nurse managers should prioritize mental health to boost PCA levels, helping nurses to maintain a positive and hopeful attitude. This will enable them to better engage with their ICU work and provide quality care (34).

### 4.4 Methods to improve nurses’ psychological capital according to research design

In terms of training system construction, centralized professional training courses can be adopted. Senior experts in the field of psychology will be invited to give lectures, and the concept of psychological capital will be systematically described, covering the core components of self-efficacy, hope, optimism, and resilience, as well as the mechanism of its influence on job performance and career satisfaction in nursing work. To stimulate the internal motivation and subjective initiative of nurses to improve psychological capital, so that nurses can apply the theories and skills they have learned to practice and effectively improve their ability to cope with high-intensity work pressure.

At the level of intervention measures, a professional psychological support team for ICU nurses can be set up in the hospital. The group was led by professional psychological counseling qualified personnel and clinically experienced senior nurses and organized regular communication and sharing activities every week. It enables them to share their work stressors, psychological distress, and coping experiences. When nurses have serious psychological stress reaction, professional psychological counselors will provide personalized psychological intervention and guidance based on psychological counseling theory and technology to enhance nurses’ psychological resilience.

In the process of task allocation, the professional ability, workload, and individual differences of nurses were fully considered, and the flexible scheduling system was implemented. According to the dynamic change law of ICU work intensity and the personal needs of nurses, the scheduling optimization algorithm was used to formulate a personalized scheduling scheme, which effectively reduced the work-life conflict and improved the job satisfaction of nurses.

### 4.5 Limitations of the study

This study relied on a literature review, questionnaire data collection, and statistical analysis to draw conclusions. However, to gain a more comprehensive and accurate analysis of psychological resilience, PCA, and burnout among ICU nurses, further research is needed. In terms of samples, the sample size was relatively small, which may affect the reliability and representativity of the results, thus limiting the generalizability of the research conclusions to a certain extent.

From the perspective of sample and potential confounding factors, this study has some improvements. The sample size is relatively small, which may affect the reliability and representativeness of the study results to some extent, and may limit the wider applicability of the study conclusions. At the same time, potential confounding factors such as work shifts, patient care volume, and differences between different hospitals were not fully considered during the study. These factors are likely to interfere with the results of the study, thereby bringing some negative effects on the accuracy of the study conclusion.

At the data level, this study has a key improvement point, that is, the lack of longitudinal data support. Analysis based only on data at a particular time point or a short period of time limits the depth of research conclusions to a considerable extent. Longitudinal data can clearly show the changes of the subjects over a long period of time, which is of great significance for revealing the dynamic correlation between variables more accurately. The lack of longitudinal data makes it difficult to determine the key question of whether the findings are due to chance at a given time or are stable over time. Therefore, subsequent related studies may try to collect longitudinal data and follow up the changes of the subjects for a long time, so as to more accurately analyze the relationship between variables and make the research conclusions more convincing. In view of the above limitations of this study, future studies should consider expanding the sample size appropriately, actively carrying out multi-center and large-sample research, and including as many subjects with different characteristics as possible. At the same time, the potential confounding factors such as work shifts and patient care volume should be fully weighed, and the influence of differences between different hospitals on the research results should be effectively controlled, so as to promote the further development of research in this field.

## 5 Conclusion

PCA fully mediates the relationship between psychological resilience and burnout among ICU nurses (35). Specifically, psychological resilience not only directly influences PCA but also indirectly affects burnout. As an internal motivator, PCA profoundly impacts burnout in nurses (36). This research represents an important advancement in international research, as it explores the interaction between psychological resilience, PCA, and burnout, a relationship that has not been comprehensively investigated in existing studies. While burnout is a consequence of sustained work-related stress, it may not be immediately apparent in the early stages of a nursing career. However, psychological resilience can be assessed and addressed early, offering opportunities for timely intervention. The findings suggest that psychological resilience and burnout have an indirect impact on one another, with resilience serving as a predictive factor for burnout, enabling early identification and intervention to improve overall wellbeing and work performance. Psychological capital appreciation played a complete mediating role between psychological resilience and job burnout. Psychological resilience does not directly affect job burnout, but effectively reduces the level of job burnout by improving the key factor of psychological capital appreciation.

## Data Availability

The original contributions presented in the study are included in the article/supplementary material, further inquiries can be directed to the corresponding author.
